# Relationship between negative coping style and fear of COVID-19 among Wuhan college students during the post-pandemic period: A moderated chain mediation model

**DOI:** 10.3389/fpsyt.2022.994685

**Published:** 2022-11-24

**Authors:** Lei Yang, Ziyun Yang, Ying Xia

**Affiliations:** ^1^Department of Psychology, Wuhan University, Wuhan, China; ^2^Institute of Adolescent Psychological Development, Zhongnan University of Economics and Law, Wuhan, China; ^3^Wuhan Wudong Hospital, Wuhan, China

**Keywords:** COVID-19, negative coping style, rumination, stress, fear of COVID-19

## Abstract

**Objectives:**

After a long-term lockdown, particularly one in which human life is at risk, negative psychological consequences are expected. In this study, we aimed to explore the cause of stress and fear of coronavirus-19 (COVID-19) among Chinese college students in Wuhan during the latest strictest lockdown.

**Methods:**

During the COVID-19 outbreak, 1,070 college students from Wuhan, aged 18–29 years, took part in an online survey. We used correlations, bootstrap tests, and other statistical analysis methods to analyze the data.

**Results:**

Negative coping style significantly positively predicted fear of COVID-19, and stress had a significant mediating effect on the relationship between negative coping style and fear of COVID-19. In addition, rumination and stress had a chain-mediating effect on the relationship between negative coping style and fear of COVID-19. Perceived social support moderated the three paths of this serial mediation model.

**Conclusion:**

Negative emotions, such as stress and fear of COVID-19, in college students are caused by both behavior (e.g., negative coping style) and cognition (e.g., rumination). Importantly, negative cognitive thinking (i.e., rumination) is often a malign consequence of a negative coping style. Thus, to improve students' mental health, students should be encouraged to engage in more positive behaviors and seek social support during periods of adherence to regular prevention and control measures.

## Introduction

The World Health Organization (WHO) declared on March 11, 2020, that the spread of the novel corona virus known as COVID-19 (in which pneumonia is a serious complication) had become a global pandemic (1). In the recommendations made by the WHO, quarantine and social distancing measures were emphasized (2). The quarantining that occurred across most countries to minimize the spread of COVID-19 significantly altered the ways in which people lived their lives. At the same time, strict compliance with quarantine regulations during the lockdown (which led to the suspension of almost all social activities) posed the risk of contributing to the onset of psychological problems, and the fear of COVID-19 posed a potential threat to public mental health (3). The Chinese government put in place unprecedented measures to combat the virus and these measures have proven to be effective. As a result, control of the pandemic has become normal in China, and people's lives and work have gradually adjusted to the new normal with regular prevention and control (e.g., intermittent closure of some public places). The Chinese media refers to this phase as the “post-pandemic period”. This period is now being explored in several Chinese studies, including mental health issues that have become apparent (4, 5).

A retrospective review of some COVID-19-related studies points to several limitations in existing research. First, some qualitative open-ended studies that only summarized the contents of coping styles of residents during the pandemic (6). Some studies explored the relationship between negative coping styles and mental health in response to societal changes, but they only performed a preliminary analysis and did not explore the underlying mechanisms (7, 8). Second, previous studies have primarily focused on the mental health of individuals during the pandemic and few studies have explored psychological changes during the post-pandemic period with widely varied lockdown. This may be because many countries have not yet entered the stage of pandemic prevention and control, therefore, exploring mental health issues during this period is prospective. Third, most current research has focused on the benefits of positive coping styles (9–11), few studies have separately explored the effects and mechanisms of negative coping styles. According to the two-dimensional theory of emotion, the effects of positive and negative emotions can work independently in some sense (12, 13). This means exploring the adverse psychological consequences of negative coping styles in isolation has its own unique significance, especially in terms of practical significance. Researches has shown that when faced with public health emergencies, Chinese college students often resort to mental avoidance or malign coping methods rather than solving problems positively (14–16), something school administrators should be aware of. Based on the above reasoning, this study focuses on exploring the relationship and mechanism between negative coping strategies and fear of COVID-19 in a post-pandemic context. Findings should be informative for other countries about to enter this phase, as well as provide theoretical inspiration for related practical interventions.

### Negative coping style and fear of COVID-19

Since it was first proposed, the conservation of resources (COR) theory has become a broad theoretical system (17–19) with distinct clinical, psychological, and theoretical features. It describes the explanatory mechanisms for behavioral expression and psychological problems. The theory assumes that the sense of stress originates from real-life situations rather than a purely subjective construction process, and emphasizes the objectivity and contextual nature of the stressor (20, 21). The COVID-19 pandemic fits with the definition of being a sudden and objectively stressful event, and the subsequent quarantine policies that limited people's behavioral motivations were contextual.

Hobfoll (21) suggested that coping with mental health problems in humans is more complex than a simple stimulus–response process, so the COR theory describes the generation of, and coping with, stress according to individual resources. The key point of the COR theory is that the accelerated loss of resources following a stressful event leads to a spiral of loss if the individual is unable to effectively resolve the stressful event and has no opportunity to organize timely compensation (21, 22). In a nutshell, COR theory views stressful events as an objective existence and people need to consume resources in order to deal with problems. Lack of resources increases the risk of coping failure, which in turn increases the psychological burden on the individual. When faced with the COVID-19 pandemic, people have used a variety of coping resources, accompanied by psychological changes. Therefore, based on COR theory, this study sought to determine the psychological mechanism underlying the relationship between college students' coping styles and fear of COVID-19.

According to the transactional model of Lazarus and Folkman (23), coping is described as an individual's cognitive or behavioral efforts to address internal or external demands in stressful situations. Coping style can be divided into two types: positive coping style is directed at changing the situation or removing the threat by using problem-solving strategies, such as redefining the problem and considering various solutions. In contrast, negative coping style is directed at changing or modifying reactions to events and involves the use of behavioral or cognitive adjustment strategies to reduce emotional stress, including problem avoidance, self-blame, and some unhealthy behaviors (e.g., smoking and drinking) (24). Studies have shown that these negative coping styles are associated with psychological distress (25, 26).

For students, limiting the scope of activities in schools was one of the lockdown measures to prevent infiltration and spread of the virus. However, quarantine can also induce numerous negative emotions, such as fear (3). It is well known that during a lockdown, individuals do not receive timely and effective resource support and adopt a passive coping approach, which will exacerbate their negative feelings about COVID-19. Previous studies have reported that negative coping predicted poor psychological consequences (27), that is, the more negative coping styles are used, the more severe the psychological problems. Furthermore, negative coping styles were found to be positively correlated with fear during the COVID-19 pandemic (28). Therefore, it was reasonable to propose H1: negative coping style will positively predict fear of COVID-19.

### Rumination

Rumination is a maladaptive form of cognitive self-reflection, the repetitive and passive recollection of negative thoughts associated with destructive events is a characteristic of rumination (29). From a COR theory perspective, individuals who are unable to stop the depletion of resources will be caught in a “stress spiral” (21, 22). In other words, individuals who adopt poor coping styles will not only fail to solve problems but this approach may even deepen their psychological distress. During the lockdown, students pass negative news to each other and this can reinforce the adverse cognitive responses. Research has shown that passive stress coping styles are associated with rumination (30) and a study with a sample of Chinese college students also indicated that negative coping style was positively correlated with each dimension of rumination (31).

Rumination threatens many components of mental health. For example, it enhances negative thinking, impairs problem-solving abilities, and interferes with support acquisition (29). A recent study in South Korea revealed that unhealthy ruminative thinking is positively associated with psychological distress (32). Furthermore, a study showed that rumination is positively associated with fear of COVID-19 (33). Given these findings, the present study hypothesized that H2: the relationship between negative coping style and fear of COVID-19 will be mediated by rumination.

### Stress

According to COR theory, individuals use certain strategies to stop the loss of resources when coping with stressful situations (22). Negative coping, as an avoidance strategy, has been found to be associated with emotional maladjustment in previous studies (34, 35). The finding that negative coping styles are positively associated with stress in the context of the COVID-19 pandemic has been widely demonstrated (36, 37). Lardier et al. (38) found 56% of students showed a high level of perceived stress during the COVID-19 pandemic in Poland, as well as negative coping styles were significantly and positively associated with stress during this time.

Previous studies have shown that a range of negative psychological problems, including stress, is associated specifically with different types of negative life events (39). There is a strong relationship between stress and fear, and stressful situations can sometimes be a source of fear (40). Based on this, it is not difficult to speculate that there is an association between stress and fear of COVID-19. Indeed, the relationship between stress and fear of COVID-19 has been demonstrated consistently (41, 42). A study among Spanish university students found that a significant positive relationship between the stress and fear of COVID-19 (43). Based on the above theory and empirical research, the present study hypothesized that H3: the relationship between negative coping style and fear of COVID-19 will be mediated by stress.

### Rumination and stress

According to the theory about rumination, rumination is not only a consequence of sudden stressors but also an antecedent of stress consequences (29). A diathesis-stress model was tested in a 6-month longitudinal study, demonstrating that rumination and stress interact to predict psychological distress (44). Another study revealed that rumination is significantly positively associated with stress (45). Set in the social ecology of COVID-19, a follow-up study on Chinese college students showed that rumination is a partial catalyst for stress consequences (46). In light of this, the present study hypothesized that H4: the relationship between negative coping style and fear of COVID-19 will be serially mediated by rumination and stress.

### Perceived social support

Perceived social support is the emotional support that individuals subjectively experience and is obtained *via* social interactions. It includes positive experiences, such as being respected and understood (47). As an effective personal resource for coping with stressful life events, social support is considered an important protective factor for mental health when individuals encounter negative life events.

**Figure 1:**
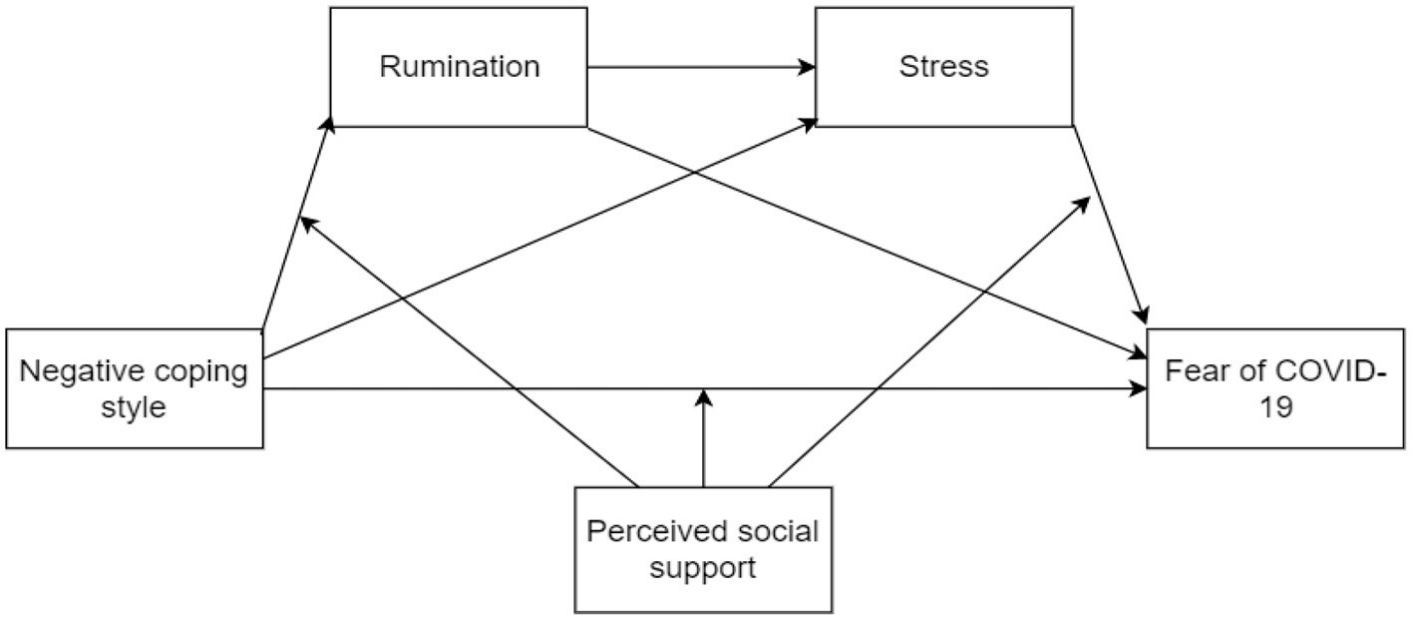
Hypothetical indirect pathways between negative coping style and fear of COVID-19, and moderating pathways of perceived social support.

A meta-analysis showed that low perceived social support is associated with post-traumatic stress disorder, and a lack of social support during or after trauma has a stronger effect than pre-trauma factors (48). The buffering model of social support states that effective social support can ameliorate the adverse psychological consequences of stress (49). A study conducted during the COVID-19 pandemic demonstrated that although individuals undergoing self-isolation had significantly higher rates of depression, those who reported high levels of social support had a 63% lower risk of depression symptoms than those with low levels of social support (50). In addition, a study of Chinese residents during the COVID-19 pandemic revealed that perceived social support effectively reduced the impact of quarantine measures; moreover, social support from family was related to lifestyle changes aimed at promoting mental health (51). Studies have also found that perceived social support is negatively associated with rumination (52). Therefore, the present study hypothesized that H5: perceived social support will moderate the serial mediation model (see Figure 1).

## Methods

### Participants

In May 2022, participants were recruited from three universities in Wuhan, China. Because COVID-19 infections had emerged in other regions, these universities were under strict management at this time. Students were restricted from leaving campus and were required to maintain social distancing. First, three of the 52 public universities in Wuhan (a comprehensive university, a polytechnic university, and a normal university) were selected during a lockdown. These three types cover a very large proportion of colleges and universities in China, so the randomly selected samples from these three universities in Wuhan are considered representative.

Then, 1,184 students were randomly selected from the students enrolled in these three universities to be survey respondents using a convenient sampling method. After removing 107 unqualified responses (e.g., failing a polygraph question and completion time <100 s), 1,070 Chinese college students (52.2% men; 47.8% women) volunteered to participate with an effective rate of 90.37%. These included 53 senior students, 53 sophomores, 440 juniors, and 524 freshmen. Participants ranged in age from 18 to 29 years, with a mean age of 19.60 years (standard deviation [*SD*] = 1.35), and 40.7% were from urban areas whereas 59.3% were from rural areas. The study was approved by the Wuhan University ethical committee. Participants completed online questionnaires using the Wenjuanxing System (Enterprise Premium Version, https://www.wjx.cn/wjxenjoy.aspx), which is an established platform providing functions equivalent to Amazon Mechanical Turk. All participants completed an informed consent form voluntarily and anonymously prior to completing the survey.

### Measures

The questionnaire included sociodemographic variables, such as sex, college year, and subjective family socioeconomic status (SES), and several other variables (as described below).

### Negative coping style

The negative coping subscale was used to measure the frequency of negative coping style use. This subscale was derived from the Simplified Coping Style Questionnaire (SCSQ) (24). The negative coping subscale is a one-dimensional, self-report instrument in which participants score eight items on a four-point Likert rating scale ranging from 0 (never) to 3 (always). A higher score indicates that individuals are more inclined to use negative coping styles or ways to minimize distress when faced with setbacks or difficulties. Example items include “when facing problems, I escape troubles by drinking, taking drugs, and smoking.” or “imagining a miracle will come, and change in the status quo.” The subscale has been shown to have high internal consistency for passive coping styles in a Chinese sample (53). The Cronbach's α in the present study was 0.80.

### Rumination

The Ruminative Responses Scale—Short Version (RRS-10) was used to measure the level of rumination. RRS-10 is a multi-dimensional scale, and includes brooding and reflection. The two-factor structure was proposed by Treynor et al. (54). The scale is a 10-item, self-report measure scored on a four-point Likert rating scale ranging from 1 (almost never) to 4 (almost always). Higher scores indicate higher rumination levels. Example items include “Why do I have problems other people don't have?” The RRS-10 has been previously used in research related to COVID-19 in the Chinese population (55). The Cronbach's α in the present study was 0.91.

### Stress

The stress subscale of the Depression, Anxiety, and Stress Scale (DASS-21) was used to measure the level of stress (56). The stress subscale is a one-dimensional instrument containing seven items (questions 1, 6, 8, 11, 12, 14, and 18). Participants rate items according to their experience on a four-point Likert rating scale ranging from 0 (did not apply to me at all) to 3 (applied to me very much). Example items include “I tended to over-react to situations.” The stress subscale has been previously used in research related to COVID-19 in the Chinese population (57). The Cronbach's α in the present study was 0.90.

### Fear of COVID-19

Participants' fear of COVID-19 was assessed using the Fear of COVID-19 Scale (FCV-19S) (58). It is a one-dimensional scale in which participants score seven items on a five-point Likert rating scale ranging from 1 (strongly disagree) to 5 (strongly agree). A higher score indicates a greater fear of COVID-19. Example items include “It makes me uncomfortable to think about coronavirus-19.” We used the Chinese version of the FCV-19S, which has been shown to have high internal consistency, reliability, and convergent and discriminant validity (59). The Cronbach's α in the present study was 0.90.

### Perceived social support

The perceived social support scale (PSSS) was used to assess the perceived support received from family, friends, and other people (60). PSSS is a multi-dimensional scale, including the dimensions of family, friends, and significant others. The scale is a validated 12-item, self-report scale, and each item is scored on a seven-point Likert scale ranging from 1 (strongly disagree) to 7 (strongly agree). A higher score indicates a higher level of perceived social support. We used the Chinese version of the PSSS, which has high internal consistency and reliability (53). The Cronbach's α in the present study was 0.97.

### Data analysis

Model 4 of the PROCESS macro for IBM SPSS Statistics Version 25.0 (61) was used to test the mediation effects described in H1 to H3, and Model 6 of the PROCESS macro was used to test the serial mediation effects described in H4 (61). To test H5, we used Model 92 (61). Sex, age, and subjective SES were controlled for as covariates, and the data were centralized for all analyses. The bootstrap method with 10,000 replicate samples was used to determine statistical significance, and 95% confidence intervals (CIs) of the indirect effects were calculated. Mediating and moderating effects were considered significant if the 95% CI did not include zero (61). All data analyses were conducted using IBM SPSS Statistics 25. The sample size was determined *via* G^*^Power 3.1 which showed that a sample of 210 participants achieved power at 0.95 to detect a medium effect for condition (*d* = 0.5, α = 0.05, two-tailed). In addition, the sample size of this study was more than 10 times the number of independent variable question items. Therefore, the sample size of this study fully met the statistical requirements.

### Common method bias tests

Harman's single-factor test was used to test for common method bias (62). All items were included in the exploratory factor analysis. Results showed that the Kaiser-Meyer Olkin (KMO) value was 0.93 and the Bartlett value was 34,184.70 (*p* < 0.001). Results of the factor analysis without rotation showed that six factors with characteristic roots >1 were generated, which explained 64.76% of the variation. The first factor explained 21.85% of the variance variation, which was below the 30% threshold. Therefore, there was no common method bias issue in our measurements.

## Results

### Preliminary analyses

Correlations, reliability, and descriptive statistics for the study variables are provided in Table 1. As expected, fear of COVID-19 was positively associated with negative coping style (*r* = 0.18, *p* < 0.001), rumination (*r* = 0.16, *p* < 0.001), and stress (r = 0.39, *p* < 0.001). Stress was positively associated with negative coping style (*r* = 0.24, *p* < 0.001) and rumination (*r* = 0.38, *p* < 0.001) and negatively associated with perceived social support (*r* = −0.20, *p* < 0.001). Rumination was positively associated with negative coping style (*r* = 0.30, *p* < 0.001). Negative coping style was positively associated with perceived social support (*r* = 0.10, *p* < 0.01).

**Table 1:** Descriptive statistics and correlations among study variables.

**Variable**	**1**	**2**	**3**	**4**	**α**	***M***	***SD***
Negative copy style	–				0.80	18.36	4.64
Rumination	0.30^***^	–			0.91	11.09	3.18
Stress	0.24^***^	0.38^***^	–		0.90	11.96	4.35
Fear of COVID-19	0.18^***^	0.16^***^	0.39^***^	–	0.90	16.93	5.84
Perceived social support	0.10^**^	−0.01	−0.20^***^	−0.01	0.97	59.40	15.27

*N* = 1070. *p < 0.05,

** *p* < 0.01,

*** *p* < 0.001.

### Mediation analyses

Results of the regression analyses are presented in Table 2. Confirming H1, we found a positive direct effect of negative coping style on fear of COVID-19 (β = 0.093, *p* < 0.01). The result suggested that students who use negative coping style more frequently reported a higher level of fear of COVID-19. In addition, negative coping style was found to be a positive predictor of rumination (β = 0.304, *p* < 0.001) and stress (β = 0.138, *p* < 0.001).

**Table 2:** Regression analysis among study variables.

**Result variable**	**Predictive variables**	***R^2^***	**β**	***t***	**95% CI**
Rumination	Negative coping style	0.099	0.304	10.395^***^	[0.247, 0.361]
Stress	Negative coping style	0.173	0.138	4.692^***^	[0.080, 0.195]
	Rumination		0.334	11.397^***^	[0.277, 0.392]
Fear of COVID-19	Negative coping style	0.172	0.093	3.130^**^	[0.035, 0.151]
	Rumination		−0.019	−0.623	[−0.080, 0.042]
	Stress		0.374	12.219^***^	[0.314, 0.434]

*N* = 1,070. The beta values are standardized coefficients.

*p < 0.05,

** *p* < 0.01,

*** *p* < 0.001.

Results of mediation analyses showed that H2 to H4 were supported. Specifically, the indirect effect of negative coping style on fear of COVID-19 via rumination was significant (β = 0.032, *SE* = 0.012, 95% CI = [0.009, 0.056]), confirming H2. The result suggested that the relationship between negative coping style and fear of COVID-19 was mediated by rumination. As well as, the indirect effect of negative coping style on fear of COVID-19 *via* stress was significant (β = 0.088, *SE* = 0.016, 95% CI = [0.059, 0.121]), confirming H3. The result suggested that the relationship between negative coping style and fear of COVID-19 was mediated by stress. Moreover, when we included two mediators in the analysis, the coefficient increased (total effect, β = 0.176, *p* < 0.001), the indirect effect of negative coping style on fear of COVID-19 *via* both rumination and stress was significant with a point estimate of 0.038 (*SE* = 0.007, 95% CI = [0.025, 0.054]). The result suggested that negative coping style could have an effect on the fear of COVID-19 via rumination and stress. Therefore, H4 was also confirmed (see Table 3).

**Table 3:** Indirect effect of intolerance of uncertainty on mental wellbeing *via* rumination and fear of COVID-19.

**Path**	**Coefficient**	**95% CI**	
		**LL**	**UL**
Negative coping style → Rumination → Fear of COVID-19	−0.006	−0.028	0.014
Negative coping style → Stress→ Fear of COVID-19	0.052	0.024	0.082
Negative coping style → Rumination → Stress → Fear of COVID-19	0.038	0.025	0.054
Total effect	0.176	0.118	0.235
Direct effect	0.093	0.035	0.151
Total indirect effect	0.084	0.050	0.120

*N* = 1,070. The beta values are standardized coefficients.

*CI* confidence interval, *LL* lower limit, *UL* upper limit.

### Moderation analyses

**Figure 2:**
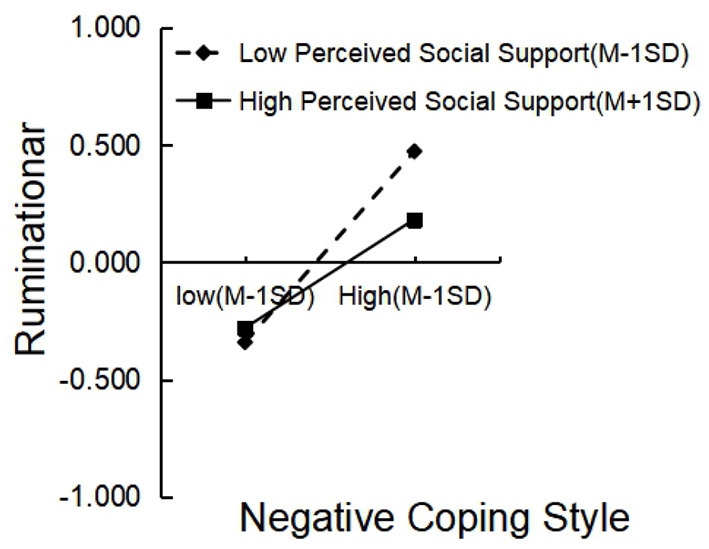
Interaction of negative coping style and perceived social support on rumination.

Consistent with H5, perceived social support moderated three paths in the chain mediation model. Specifically, perceived social support moderated the relationship between negative coping style and rumination. A follow-up simple slope analysis revealed that negative coping style predicted rumination for participants with high (+1 SD) perceived social support (β = 0.232, *t* = 6.422, *p* < 0.001), and negative coping style significantly predicted rumination with low (−1 SD) perceived social support (β = 0.408, *t* = 10.234, *p* < 0.001; Figure 2).

**Figure 3:**
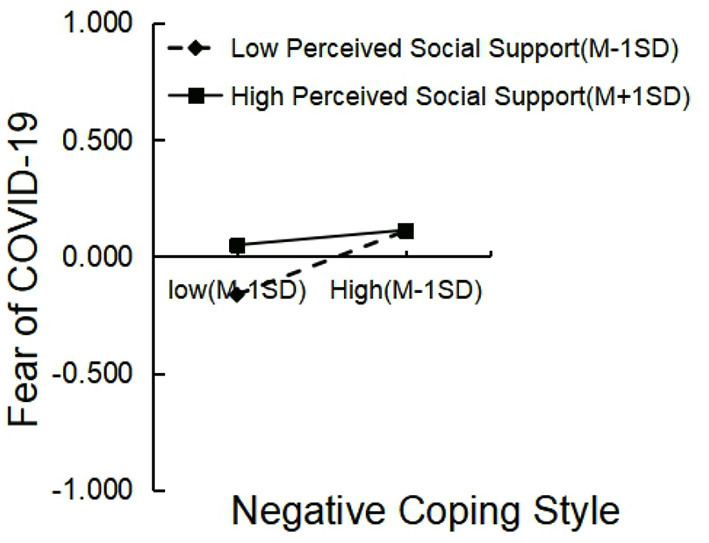
Interaction of negative coping style and perceived social support on fear of COVID-19.

Additionally, perceived social support moderated the relationship between negative coping style and fear of COVID-19. A follow-up simple slope analysis revealed that negative coping style did not predict fear of COVID-19 for participants with high (+ 1 SD) perceived social support (β = 0.032, *t* = 0.871, *p* = 0.384), whereas negative coping style predicted fear of COVID-19 for those with low (– 1 SD) perceived social support (β = 0.137, *t* = 3.230, *p* < 0.01; Figure 3).

**Figure 4:**
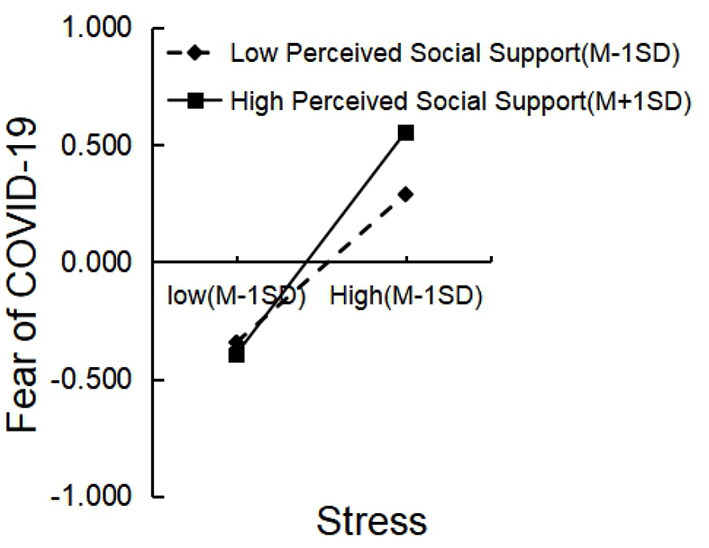
Interaction of stress and perceived social support on fear of COVID-19.

Finally, Perceived social support moderated the relationship between stress and fear of COVID-19. A follow-up simple slope analysis revealed that stress predicted fear of COVID-19 for participants with low (−1 SD) perceived social support (β = 0.316, *t* = 7.990, *p* < 0.001), and stress significantly predicted fear of COVID-19 for those with high (+ 1 SD) perceived social support (β = 0.475, *t* = 11.582, *p* < 0.001; Figure 4).

**Figure 5:**
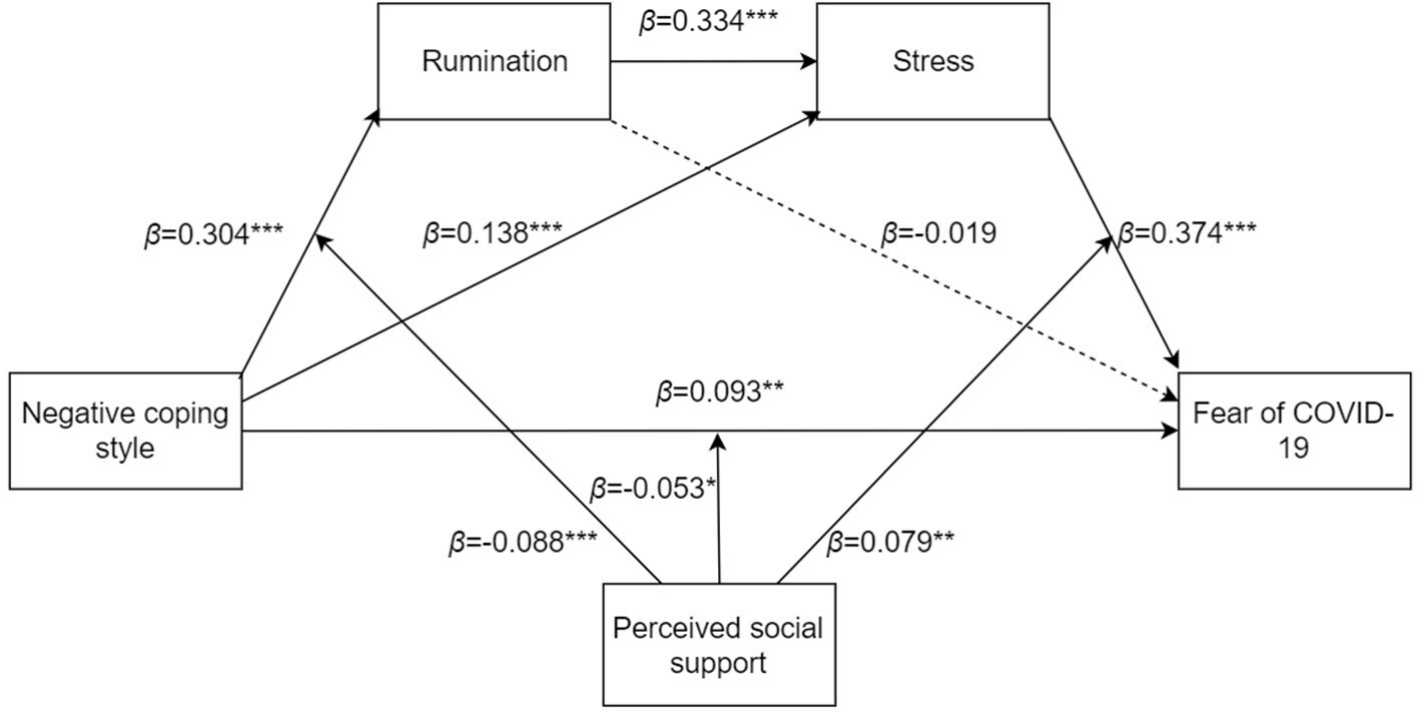
The moderated mediation model of negative coping style, rumination, stress, fear of COVID-19, and perceived social support.

To summarize, the results of this study indicated that negative coping style positively predicted fear of COVID-19, and there was a significant indirect effect of negative coping style on fear of COVID-19 *via* stress. The association between coping style and fear of COVID-19 was partially mediated by high levels of both rumination and stress, and perceived social support played a moderating role in the serial mediation model (see Figure 5).

## Discussion

When this study was conducted, the COVID-19 pandemic had entered the normalization management stage in China. The COVID-19 pandemic was an obvious stressor and had induced changes in the physical and mental behavior of many people. This coupled with the impact of quarantine policies that limited individuals' access to resources, and the sense of uncertainty and loss of control, impacted psychological health (63). Therefore, closely examining the negative psychological ramifications of the pandemic is crucial, and prompted us to explore the relationships among negative coping style, rumination, stress, and fear of COVID-19. The results of the study confirmed our hypotheses, which we discuss next.

### Negative coping style is relevant to fear of COVID-19

The results of this study indicated that negative coping style positively predicted fear of COVID-19, which was consistent with the results of previous studies, and H1 was verified. According to the COR theory, the generation of stress is not a purely internal psychological process but rather, a process of continuous interaction between the individual and the situation (22). In response to the threatening event that was the COVID-19 pandemic, people took various means to protect themselves. For instance, in the United Kingdom, various positive and adverse coping strategies were used simultaneously by individuals (6). The crux of the matter lies in that coping styles play a pivotal role in mental health, particularly during adaptation to stress caused by COVID-19 (64). For example, research conducted in Australia showed that behavioral disengagement led to higher levels of depression (7). The results of this study also provided supporting evidence. This study revealed that Chinese college students who applied a more negative coping style increased their mental health problems, such as fear of COVID-19. Specifically, students who applied more negative coping strategies had higher levels of rumination and stress, and this in turn increased their fear of COVID-19.

### The mediating effect of rumination and stress

In line with H2 and H3, the results indicated that negative coping styles influenced fear of COVID-19, fueled by rumination. Meanwhile, stress was found to mediate the relationship between negative coping style and fear of COVID-19. Importantly, the main hypothesis (H4) was confirmed. The results of this study support the serial mediation model, which indicated that rumination and stress play mediating roles in the relationship between negative coping style and fear of COVID-19.

During the quarantine period of the COVID-19 pandemic, individuals were forced to turn to negative coping styles because of limiting objective factors, such as the restriction of movement to school or home, which increased the risk of developing psychological problems (65). In general, a positive coping style involves proactively taking constructive action. In contrast, those who adopt negative coping styles try to avoid stress as a way to minimize distress and focus on negative thoughts (66). Because students were only able to visit a few fixed locations during the normalization stage of the COVID-19 pandemic, their external activities were disrupted, and contact with the outside world decreased substantially, which exhausted their coping strategies, which possibly turned to being progressively more negative. Examples include avoidance strategy and the use of unhealthy means to solve problems (such as drinking or smoking).

However, in reality, these coping measures do not solve the problem but excessively deplete the individual's psychological resources. In such situations, individuals likely ruminated about and analyzed COVID-19-related events. Studies did confirm that young people spent considerable time thinking about the COVID-19 pandemic (67). However, previous studies have provided evidence that dysphoric rumination leads students to recall more bad situations or memories than good ones (68, 69). Moreover, repeated thinking about a catastrophic event results in the enhancement of negative self-evaluations that are observed clinically (70). According to COR theory, stress increases when individuals run out of psychological resources but fail to solve problems successfully (21, 22). Therefore, students with higher levels of rumination may believe that they are less capable of problem solving, and then perceive more stress.

Further, along with unresolved problems, students' feelings of helplessness can lead to fear under COVID-19 lockdown. As previous studies have shown, stress leads to the development of fears (40). Consequently, through reinforcement by rumination and stress, students who adopt a negative coping style can become more fearful of the COVID-19 pandemic.

### The moderating effect of perceived social support

Numerous studies conducted during the COVID-19 pandemic have shown that social support has a significant impact on mental health outcomes (50, 71, 72). We found that perceived social support moderated three pathways of the model, namely, the relationships between negative coping style and rumination, negative coping style and fear of COVID-19, and stress and fear of COVID-19. In the two pathways related to negative coping style, the moderating role of social support is not difficult to understand. Perceived social support contributes to health-promoting behaviors and was shown to have a particularly significant impact on compliance with stay-at-home orders during the COVID-19 outbreak (73). Thus, in the pathway involving a negative coping style, the deleterious effects may be enhanced under low perceived social support conditions. Specifically, individuals who received sufficient coping resources initially were likely to appropriately manage the stress according to the COR theory (22). Conversely, in the absence of social support, it is difficult for individuals to adopt active problem-centered coping strategies, which can lead to the production of more passive coping measures, such as drinking, avoidance, and fantasy in lockdown. However, when these approaches prove ineffective, individuals become trapped in painful reflections and become more fearful of COVID-19.

Surprisingly, for the relationship between stress and fear, the harmful effect was amplified in high perceived social support conditions. We speculate that this may reflect a special psychological state that arose during the post-pandemic period. The echo chamber effect describes how information in a group gradually becomes homogenized, and discussions can lead to the increase of irrational emotions among group members (74, 75). During the long-term lockdown, a cocoon of information was formed within schools, so students moving within the school boundaries were readily exposed to negative information from those around them when seeking social support, for example, when confiding in roommates. In such cases, students who applied a strategy to seek out surrounding social support resources in high-stress situation often received negative feedback about COVID-19, creating a vicious cycle and echo chamber effect. This eventually would lead to even less hope and increased fear of COVID-19. Of course, this is only a preliminary inference combining the current study results and our assessment of the post-pandemic situation, and more studies are needed to verify and explore the mechanisms involved.

### Practical applications

Given these findings, this research contributes to the development of practical applications. First, administrative departments of universities could develop programs to reduce fear of COVID-19 that can be implemented online or offline, such as photography-based interventions (76), and administrative departments also can host various on-campus events, such as yoga/meditation group counseling, campus concerts, movies, and other interesting collective events. These activities can help students break out of information cocoons to prevent or reduce their psychological fear. Second, when giving advice, college counselors should encourage students to confide in them from the perspective of seeking social support (e.g., talking to more experienced teachers or family members) rather than talking to classmates who also harbor negative emotions, as this may instead deepen the negative rumination of the person seeking help.

The present study mainly explored and demonstrated the psychological crisis brought about by negative coping style during the post-pandemic period. This serves as a caution significance to school administrators. So schools could encourage students to engage in positive coping on their own, such as listening to music, reading books, and taking daily exercise. In addition, because college students have access to various types of information during quarantine *via* social media, social media channels, such as Wechat, Douyin, and Weibo, may be used to spread negative messages. For this reason, along with academic instruction, educators need to remind students to carefully screen information and obtain useful information from online sources.

### Limitations and prospects

This study has several limitations. First, although the sample size was sufficient, the cross-sectional design did not enable us to determine causal relationships among variables. Thus, associations should be explored using a longitudinal design to verify the circular model, whereby during public health emergencies, maladaptive coping styles can lead to negative psychological consequences, which in turn can strengthen maladaptive coping styles and cognitions. Second, the present study used self-report measures. Future studies could explore variables related to COVID-19 and other mental health outcomes using alternative methods, such as the empirical sampling method and/or behavior observation to overcome the subjective biases of self-report measures. Additionally, the study was conducted on a sample of college students. During isolation, the movement of the general population may be more restricted than that of students; thus, our findings may not generalize to the general population. Moreover, the present study presented some interesting results, such as the positive correlation between negative coping style and perceived social support, which needs to be verified further, although the same conclusion has been reached by previous studies (77, 78). We also found a discrepancy in the moderation of perceived social support across different pathways, which may predict complex changes in the psychology of individuals in the particular context of the COVID-19 post-pandemic, and therefore, more explanations are expected from future related studies.

## Conclusions

We showed in a Wuhan college student sample using serial multiple mediational analyses that rumination and stress play a chain-mediated role in the relationship between negative coping style and fear of COVID-19. Therefore, we suggest that negative coping styles promote negative cognition, such as rumination, and these negative behaviors and emotions give rise to negative psychological outcomes (such as stress and fear of COVID-19) in those in isolation during the COVID-19 post-pandemic. The observed chain pathway provides a theoretical reference for mental health interventions during the normalization stage of COVID-19 prevention and control. In addition, perceived social support served as a moderating variable that could help protect the individuals from the negative effects of negative coping style and rumination, and also serve as a signal of psychological crisis in the particular case of quarantine.

## Data availability statement

The raw data supporting the conclusions of this article will be made available by the authors, without undue reservation.

## Ethics statement

The studies involving human participants were reviewed and approved by the Ethics Committee of the Department of Psychology, School of Philosophy, Wuhan University. The patients/participants provided their written informed consent to participate in this study.

## Author contributions

LY generated the idea, designed study, and wrote the manuscript. ZY and YX participated in the data collection and supervised the study. All authors have contributed to and have approved the final manuscript.

## Funding

This study was supported by the Fundamental Research Funds for the Central Universities, Zhongnan University of Economics and Law (Grant No. 2722022DS002) and Youth Project of Wuhan Municipal Health Commission (Grant No. WX18Q30).

## Conflict of interest

The authors declare that the research was conducted in the absence of any commercial or financial relationships that could be construed as a potential conflict of interest.

## Publisher's note

All claims expressed in this article are solely those of the authors and do not necessarily represent those of their affiliated organizations, or those of the publisher, the editors and the reviewers. Any product that may be evaluated in this article, or claim that may be made by its manufacturer, is not guaranteed or endorsed by the publisher.
